# Feature Matching of Microsecond-Pulsed Magnetic Fields Combined with Fe_3_O_4_ Particles for Killing A375 Melanoma Cells

**DOI:** 10.3390/biom14050521

**Published:** 2024-04-26

**Authors:** Yan Mi, Meng-Nan Zhang, Chi Ma, Wei Zheng, Fei Teng

**Affiliations:** 1 State Key Laboratory of Power Transmission Equipment Technology, School of Electrical Engineering, Chongqing University, Chongqing 400044, China; zhangmengnan@cqu.edu.cn (M.-N.Z.); machi@cqu.edu.cn (C.M.); zhengwei_0406@stu.cqu.edu.cn (W.Z.); 2 Chongqing Key Laboratory of Translational Research for Cancer Metastasis and Individualized Treatment, Chongqing University Cancer Hospital, Chongqing 400030, China; fei.teng@cqu.edu.cn

**Keywords:** microsecond-pulsed magnetic field, Fe_3_O_4_ particles, feature matching, melanoma cells

## Abstract

The combination of magnetic fields and magnetic nanoparticles (MNPs) to kill cancer cells by magneto-mechanical force represents a novel therapy, offering advantages such as non-invasiveness, among others. Pulsed magnetic fields (PMFs) hold promise for application in this therapy due to advantages such as easily adjustable parameters; however, they suffer from the drawback of narrow pulse width. In order to fully exploit the potential of PMFs and MNPs in this therapy, while maximizing therapeutic efficacy within the constraints of the narrow pulse width, a feature-matching theory is proposed, encompassing the matching of three aspects: (1) MNP volume and critical volume of Brownian relaxation, (2) relaxation time and pulse width, and (3) MNP shape and the intermittence of PMF. In the theory, a microsecond-PMF generator was developed, and four kinds of MNPs were selected for in vitro cell experiments. The results demonstrate that the killing rate of the experimental group meeting the requirements of the theory is at least 18% higher than the control group. This validates the accuracy of our theory and provides valuable guidance for the further application of PMFs in this therapy.

## 1. Introduction

The combination of MNPs and magnetic fields to treat cancer by exerting magneto-mechanical force (MMF) on cancer cells is an emerging method known as magneto-mechanical therapy (MMT) [1,2,3,4]. Compared with traditional methods such as surgery [5], radiotherapy [6], and chemotherapy [7], this novel method offers advantages including minor side effects [3], the absence of suffering, and the preservation of healthy tissue and organs. Consequently, it has become a hot topic in current research.

**Figure 1:**
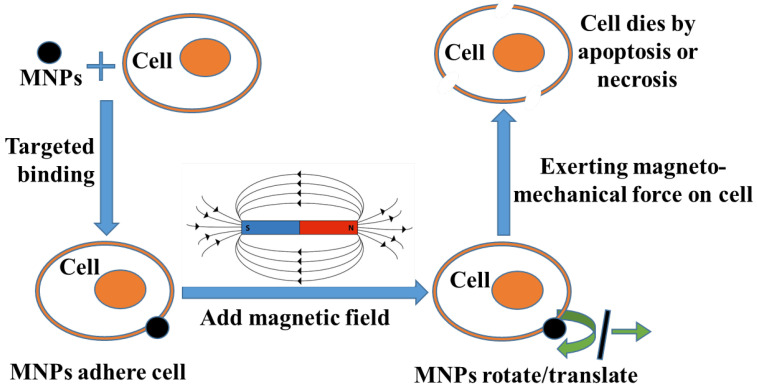
Schematic diagram of MMT.

The main process and principle of this method are shown in Figure 1. Initially, MNPs are targeted to cancer cells. Subsequently, an external magnetic field is applied that causes the MNPs to rotate or translate, thereby exerting MMF on the cancer cells. Ultimately, the cancer cells undergo apoptosis [8,9,10] or necrosis [1,11,12,13] under the force. The key aspect in this process lies in effectively exerting MMF on the cancer cells. Three factors determine the efficacy of this force: MNPs, magnetic fields, and the matching between them.

Magnetic fields play a significant role in MMT, and therefore, specific magnetic fields have been developed for MMT with distinct features. For instance, Lunov et al. developed a PMF generator with a high intensity (up to 8 T) [14,15] and short pulse width (15 µs). Additionally, a generator was utilized in order to drive superparamagnetic iron oxide nanoparticles in lysosomes, thus inducing apoptosis. MNPs are similarly important in MMT; Cheng et al. synthesized Zn-doped iron oxide nanocubes for MMT [16,17]. The combination of nanocubes and a rotating magnetic field generated by two NdFeB magnets can effectively induce lysosomal damage in cells. Nikitin et al. used four sizes of MNP (5 nm, 14 nm, 27 nm, and 99 nm) to explore the influence of MNP properties on MMT [18]. The results show that the rotational–vibrational movement of MNPs (14 nm), induced by exposure to a magnetic field, caused the strongest mechanical destruction of oligonucleotide duplexes. Specifically, magneto-mechanical effects do not exhibit a positive correlation with the size of MNP, potentially due to the magnetic−dipole interaction. In addition, it is essential to investigate both the properties of magnetic fields and MNPs simultaneously. Wang et al. developed a biaxial magnetic field and used MNPs with different aspect ratios for MMT [13,19]. The results demonstrated that the effects of magnetic fields with biaxiality were better than those with uniaxiality, and small MNPs with large aspect ratios were better than others.

These articles show that magnetic fields, MNPs, and matching between them all have an effect on MMT, and this effect is not monotonous. Therefore, determining their parameters becomes crucial, especially when they possess both favorable and unfavorable properties. Otherwise, their full potential may not be harnessed to maximize therapeutic efficacy.

Compared with the traditional magnet-generated magnetic fields, PMFs offer advantages such as high upper limit of intensity, closing at any time, easy adjustment of parameters, and low environmental requirements, thus presenting broad prospects for application in MMT. However, PMFs also suffer from the drawback of narrow pulse width, which can cause it to act on MNPs for too short a time in each cycle. This may result in the MNPs being unable to rotate or translate fully, making them unable to exert MMF on the cancer cells, ultimately weakening the therapeutic efficacy. This could explain why there are relatively few articles focusing on PMF applications in MMT compared to other types of magnetic fields with distinct features [3].

Consequently, in order to promote the application of PMFs in MMT and maximize its advantages, based on the issue of narrow pulse width, a feature-matching theory between PMFs and MNPs is proposed, including the matching of the following three parts:

1. MNP volume and the critical volume of Brownian relaxation;
2. MNP relaxation time and pulse width;
3. MNP shape and the intermittence of PMF.

In order to study this theory, a microsecond-pulsed magnetic field (μs-PMF) generator was developed, and four kinds of MNPs were selected for an in vitro cell experiment to verify the correctness of it and the effectiveness of the PMF in MMT.

## 2. Materials and Methods

### 2.1. Cell Culture

The cancer cells used in this article were human malignant melanoma cells (A-375), purchased from Procell Life Technology Co., Ltd. in Wuhan, China. Melanoma cells are a common type of human-derived cancer cell with a high degree of malignancy. They can be used for biomedical experiments, such as three-dimensional cell cultures, high-throughput screening, etc.

A375 cells were cultured in Duchenne’s Modified Eagle medium (DMEM, Biosharp, Hefei, China) containing 10% fetal bovine serum (FBS, Gibco, New York, NY, USA) and 1% penicillin-streptomycin (PS, Gibco, New York, NY, USA). The cells were cultured in an incubator at a temperature of 37 °C, a CO_2_ volume fraction of 5%, and with a humid environment. When cancer cells grow to cover 80% of the bottom area of the culture bottle, they can be digested from the bottle for passage or MMT experiment.

### 2.2. μs-PMF Generator

We previously developed a high dB/dt (the time rate of the change of magnetic flux density) nanosecond-pulsed magnetic field (ns-PMF) generator [20,21]. However, the ns-PMF generator has limitations including a narrow pulse width (hundreds of nanoseconds) and high temperature. The narrow pulse width hindered the full response of MNPs to the ns-PMF in each cycle, while the high temperature compromised the non-thermal advantages of MMT. Consequently, the ns-PMF generator was not suitable for the MMT using MMF to kill cancer cells. In order to address these issues, a new μs-PMF generator was developed in this study.

The circuit principle of the new μs-PMF generator is essentially identical to that of the previous ns-PMF generator [20,21], thus requiring minimal elaboration. Subsequently, a detailed account is provided on how the new generator addresses the two issues.

The first issue pertains to the narrow pulse width, and two measures were implemented to address this. One measure was used to increase the capacitance of the energy storage capacitor. Equation (1) shows the formula for the discharge time constant of an energy storage capacitor. According to the formula, the larger the capacitance value, the greater the discharge time constant, that is, the higher the limit of the pulse width. Therefore, an energy storage capacitor with a larger capacitance value was selected. Compared to the previous generator, the capacitance value increased from 10 μF to 100 μF. Another measure was to adjust the program of the field-programmable gate array (FPGA). By further adjusting the program, the conduction time of the Insulated Gate Bipolar Transistor (IGBT) solid-state switch assembly in the discharge circuit was extended, thereby extending the duration of the pulse current, that is, the pulse width was increased. After improvement, compared to the ns-PMF generator, the pulse width increased from hundreds of ns to tens of μs.

(1) τ=RC

where *R* is the resistance in the discharge circuit and *C* is the capacitance of the energy storage capacitor.

The excessive temperature of the inductive coil was the second issue. It was addressed by decreasing heat generation and enhancing heat dissipation. In terms of reducing heat generation, the frequency of the generator’s output current was reduced from a few hundred kHz to a few Hz by adjusting the control program of the FPGA. In terms of increasing heat dissipation, a semi-conductor cooling module (SCM) was fixed to the back of the coil to cool it down. This study also used the technology of double-layer printed circuit board (PCB). The inductive coil was placed on the top layer of the PCB, while a large area of copper coating was laid on the bottom layer of the PCB in order to quickly transmit the heat of the coil. Based on the above, the high temperature problem of the generator was solved. When μs-PMF is working, the temperature of the cell solution is always controlled below 35 °C.

**Figure 2:**
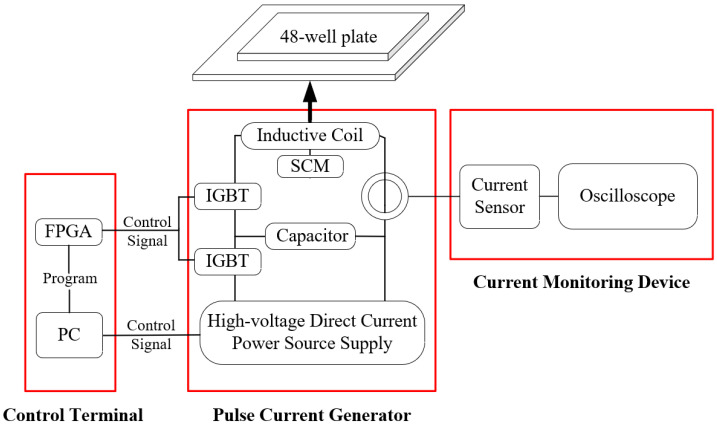
Structure of the μs-PMF generator.

**Figure 3:**
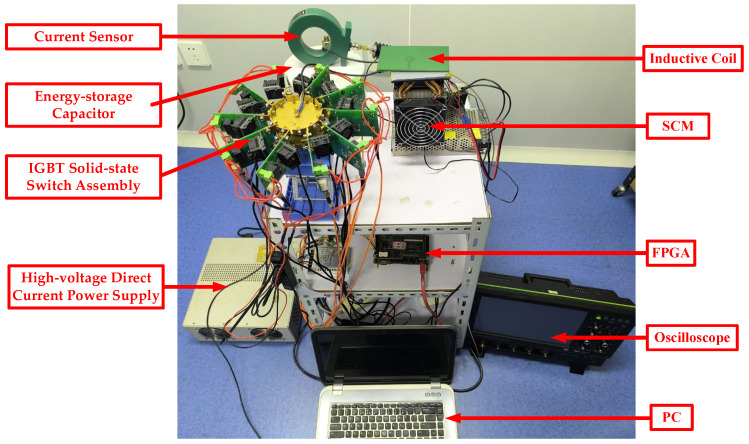
Photograph of the μs-PMF generator.

The overall structure of the new generator is shown in Figure 2 and a photograph of it is shown in Figure 3. The device consists of three parts: control terminal, pulse current generator, and current monitoring device. The control terminal was used to regulate the parameters of μs-PMF, including the FPGA and a personal computer (PC). A pulse current generator was used to generate the μs-PMF, including a high-voltage direct current power supply (SLM3P300, Spellman, New York, NY, USA), an IGBT solid-state switch assembly (IXEL40N400, IXYS, San Francisco, CA, USA), an energy storage capacitor (MMJ5KV-100 μF, Xi’an Farah, Xi’an, China), inductive coil, and an SCM (TEC1-11104, Guangdong Fuxin Technology Company Ltd., Guangzhou, China). A current monitoring device was used to record and monitor the current waveform, and included a current sensor (Model 110, Pearson Electronics, Palo Alto, CA, USA) and an oscilloscope (HDO6034A, Teledyne LeCroy, Chestnut Ridge, NY, USA).

In addition, by addressing the issue of excessive coil temperature, the risk of the inductive coil burning out due to overheating was significantly diminished. Consequently, the amplitude of the current was further increased, generating a stronger magnetic field. The number of IGBT solid-state switches in the discharge circuit was increased from six to ten. The theoretical current withstand value increased from 1200 A to 2000 A. At the same time, in order to ensure electrical safety, a certain margin should be left for the actual current. After testing, compared with the ns-PMF generator previously used, the amplitude of the current increased from 600 A to 1640 A (corresponding to an increase in magnetic flux density from 500 mT to 1.22 T).

**Figure 4:**
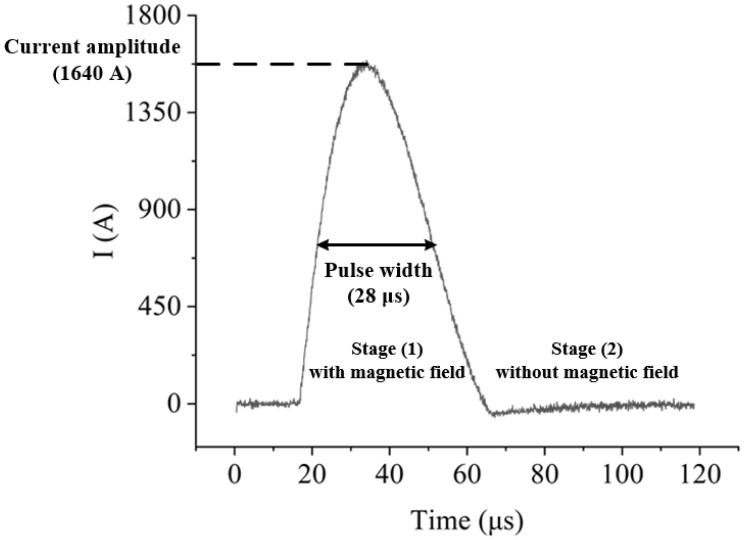
Single pulse current waveform.

The output current waveform of the new generator, that is, the μs-PMF waveform is shown in Figure 4. The relevant parameters were a current amplitude of 1640 A, a pulse width of 28 μs and a frequency of 1 Hz. In addition, it can also be seen that the μs-PMF has the features of periodicity and intermittence. Each cycle can be divided into two stages, namely, stage (1) with magnetic field, and stage (2) without magnetic field; the two stages alternate.

### 2.3. MNPs

In this section, the relevant parameters of the MNPs (including type, shape, and volume) were determined according to the parameters and features of the μs-PMF, and the matching theory between them is proposed.

#### 2.3.1. Type of MNPs

Due to their easy synthesis and good magnetic properties [22], Fe_3_O_4_ nanoparticles (IONPs) have been widely used in biomedical fields, including in magnetic resonance imaging [23,24,25,26], cell sorting [27], drug delivery [28,29], etc. [30,31,32]. Although IONPs have a certain toxicity, their biocompatibility can be improved by modifying their surfaces with polyethylene glycol (PEG) [33,34]. In addition, the layer of PEG can also weaken the interaction between IONPs and enhance their dispersion in solution, so the influence of the interaction between IONPs on MMT is not considered in this paper. Therefore, IONPs with PEG-modified surfaces were selected for MMT.

#### 2.3.2. Volume of MNPs

MNP volume was determined based on matching theory and MMF, including the following three parts:

1. Matching between MNP volume and the critical volume of Brownian relaxation;
2. Matching between MNP relaxation time and the pulse width;
3. The influence of MNP volume on MMF.

1. Matching between MNP volume and the critical volume of Brownian relaxation

**Figure 5:**
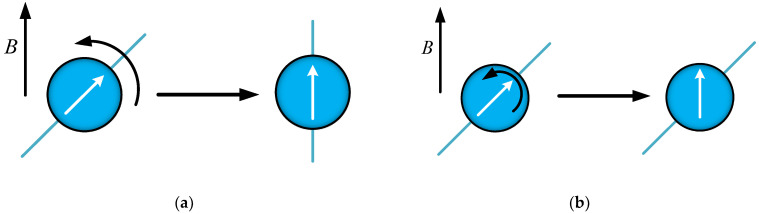
Schematic diagram of magnetic relaxation. (**a**) Magnetic moment and particle rotate as a whole (Brownian relaxation); (**b**) magnetic moment rotates within a particle (Neel relaxation).

**Figure 6:**
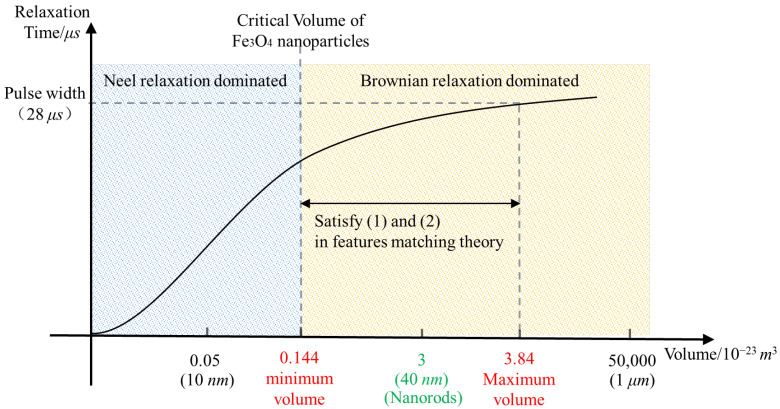
Schematic diagram of relaxation time (without considering interaction between MNPs).

As shown in Figure 5, there are two relaxation mechanisms for MNPs [35,36]. The first is the Brownian relaxation mechanism; Figure 5a shows the magnetic moment (white arrow) and particle rotate as a whole under the action of the magnetic field. The second is the Neel relaxation mechanism, as shown in Figure 5b, where the magnetic moment rotates within a particle under the action of magnetic field, while the particle itself has no rotational motion. The relaxation mechanisms of MNPs are affected by many factors, such as MNP volume, the viscosity of the environment, magnetic field, etc., which is a complicated subject. With reference to the relevant literature [37,38], a simplified research method was adopted in order to analyze the relaxation mechanism, only considering the effects of magnetic anisotropy caused by MNP size and the viscosity of environment. Strongly anisotropic MNPs were kept in a viscous carrier liquid because MNPs disperse in mediums with a viscosity close to that of water. Therefore, the particle volume plays a decisive role in the relaxation mechanism when the viscosity of the solution has been determined. As shown in Figure 6, there is a critical volume: when MNP volume is larger than that, Brownian relaxation dominates; when MNPs volume is smaller than that, Neel relaxation dominates. By consulting the relevant literature, it can be known that the critical volume of IONPs is about 1.44 × 10^−24^ m^3^ [35,36,39].

2. Matching between MNP relaxation time and the pulse width

Although the μs-PMF offers the advantages of a high upper limit of intensity, closing at any time, and so on, it suffers from the drawback of a narrow pulse width (28 μs). This makes the time of magnetic field acting on MNPs very limited in each cycle. Consequently, there is a risk that the MNPs will not finish their magnetization rotation within a single pulse, ultimately rendering them unable to exert MMF on cancer cells.

Therefore, in order to guarantee complete rotation of the MNPs during each cycle, the second point of the theory was proposed: matching between MNP relaxation time and the pulse width. That is, the time for the MNPs to complete a rotational motion in response to the magnetic field (i.e., the relaxation time of the Brownian relaxation) should be less than the pulse width.

The formula for calculating the Brownian relaxation time is shown in Equation (2) [40], and indicates that the Brownian relaxation time increases with the increasing volume of MNPs. The pulse width is about 28 μs. Taking this value as the limit into Equation (2), it was calculated that the maximum volume of IONPs that can complete the Brownian relaxation in a cycle is about 3.84 × 10^−23^ m^3^ (hydrodynamic size).

(2) $$t_{B}=\frac{3\eta_{c}V}{k_{0}T}$$

where *η_c_* is the viscosity of the solution, taken as the viscosity of the aqueous solution at room temperature (20 °C), which is approximately 1.0 × 10^−3^ Pa∙s; *V* is the hydrated size of MNPs; *k*_0_ is the Boltzmann constant, taken as 1.38 × 10^−23^ J/K; and *T* is the ambient temperature, taken as 298 K.

3. The influence of MNP volume on MMF

Equation (3) represents the torque exerted by a magnetic field on MNPs [41,42]. According to the formula, an increase in the volume of MNPs leads to a corresponding increase in the torque they are subjected to, subsequently resulting in a stronger MMF exerted on cancer cells and a better killing effect. Therefore, the volume should be as large as possible. However, it cannot be unlimitedly large; according to the analysis in part (2), the maximum volume was determined to be 3.84 × 10^−23^ m^3^.

(3) L=MVBsinθ

where *M* is the saturation magnetization of the particle, approximately 50 emu/g [43,44,45]; *V* is the particle volume; *B* is the magnetic flux density; and *θ* is the angle between the long axis of the particle and the direction of the magnetic field.

To sum up, the volume should be in the range of 1.44 × 10^−24^ m^3^ ~ 3.8 × 10^−23^ m^3^, and as large as possible. To leave a certain margin for the pulse width, it was finally determined to be about 3 × 10^−23^ m^3^, which is roughly equivalent to a nanosphere with a diameter of 40 nm. As mentioned above, the surfaces of IONPs were modified with PEG, which improved their biocompatibility and weakened the interaction between them. The relevant literature shows that after PEG modification, the hydrodynamic size will increase by 5~10 nm compared with the core size of IONPs [46]; in other words, the thickness of the modified layer was about 2.5~5 nm. This is not very thick compared to the size determined above. Therefore, according to Equation (4) [47], there was not much difference between the hydrodynamic size and the core size of IONPs, which will not cause the difference in requirements of relaxation time on the pulse width in the order of magnitude. Therefore, in order to facilitate customization of IONPs, the calculated size (hydrodynamic size) was used as the core size. That is, the main volume of an MNP was considered equal to its hydrodynamic volume in Figure 6.

(4) $$V_{H}={\left(1+\delta /R\right)}^{3}V_{M}$$

where *V_H_* is the hydrodynamic size of the particle; *V_M_* is the core size of the particle; δ is the thickness of the modified layer; and *R* is the radius of the core size.

#### 2.3.3. Shape of MNPs

Using the volume determined in Section 2.3.2, the magnetization rotation of MNPs can be completed within each cycle of μs-PMF. Furthermore, due to the periodicity and intermittence of the μs-PMF, the MNPs repeat this rotation. Related articles have demonstrated that the rotation of rod-shaped particles can have a stronger effect on cells than that of spherical particles [48,49,50]. Moreover, due to the shape asymmetry and magnetic anisotropy of rod-shaped particles, their rotation in a magnetic field is more significant than that of spherical particles [41,51]. Therefore, it was determined that MNPs should be rod-shaped, leading to the third point of the theory: matching between MNPs shape and the intermittence of μs-PMF.

**Figure 7:**
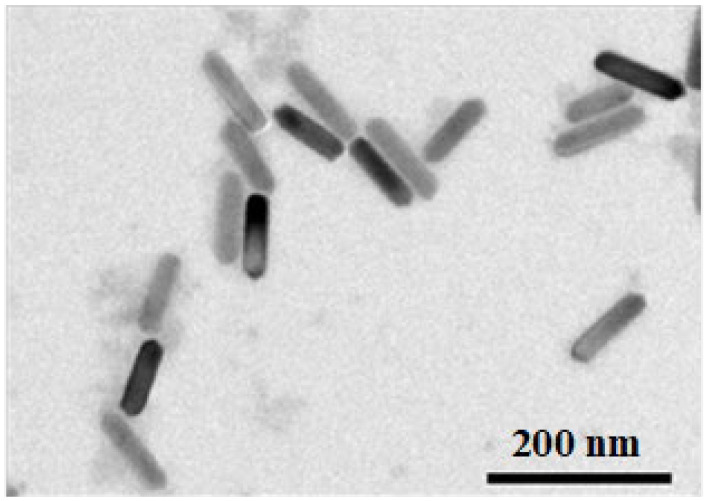
TEM of the Fe_3_O_4_ nanorods.

Based on the above analysis, the type, volume, and shape of the MNPs were determined—Fe_3_O_4_ nanorods with a volume of about 3 × 10^−23^ m^3^. The nanorods were customized in Xi’an Rui’xi Biotechnology Co., LTD., Xi’an, China. Some articles have shown that folic acid (FA) is overexpressed on the surfaces of many kinds of cancer cells (including melanoma cells) [52,53], and some of the relevant literature has utilized FA to enhance the ability of MNPs or drugs to target A375 cells [54,55]. Therefore, in order to improve the biocompatibility of the MNPs and their ability to target cancer cells, polyethylene glycol–folic acid (PEG–FA) was modified on their surfaces. Figure 7 shows a transmission electron microscope (TEM) image of the nanorods, with a length of about 100 nm and a diameter of about 20 nm. According to our calculations, the volume is approximately 3 × 10^−23^ m^3^, capable of meeting the above requirements.

**Figure 8:**
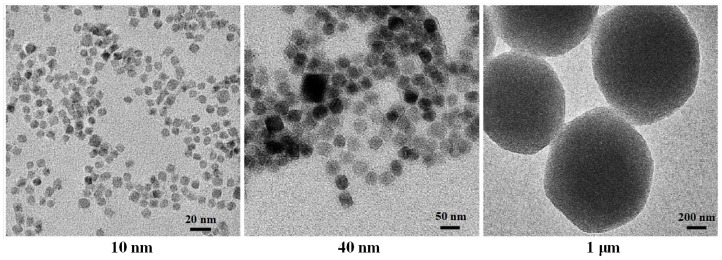
TEM of the Fe_3_O_4_ nanospheres.

In addition, in order to further demonstrate the matching theory, three additional types of IONPs were also customized in Xi’an Rui’xi Biotechnology Co., LTD., Xi’an, China. They were Fe_3_O_4_ nanospheres with diameters of 10 nm, 40 nm, and 1 μm, respectively. Their surfaces were also modified with PEG–FA. The TEM images of these three nanospheres are shown in Figure 8. Among them, the nanospheres with a diameter of 10 nm did not satisfy the first point of the theory, matching between the MNP volume and the critical volume of Brownian relaxation; the nanospheres with a diameter of 1 μm did not satisfy the second point of the theory, matching between the relaxation time and the pulse width; and the nanospheres with a diameter of 40 nm did not satisfy the third point of the theory: matching between the MNP shape and the intermittence of the μs-PMF.

### 2.4. Simulation Method for Magnetic Field Distribution

The μs-PMF was generated by the flow of a pulse current through an inductive coil, which was constructed as an Archimedean spiral coil. In order to understand the spatial distribution and strength of the μs-PMF excited around the coil, COMSOL Multiphysics 6.0 was used to model and simulate the magnetic field [20].

During exposure to the μs-PMF, the cancer cells were incubated in the 48-well plate for 36 h and adhered to the bottom of the plate. The thickness of the bottom was 1 mm. During the treatment processing, the bottom was tightly attached to the Archimedean spiral coil. Therefore, the magnetic field was parallel to and 1 mm away from the coil around the cancer cell, which also needed to be simulated.

**Figure 9:**
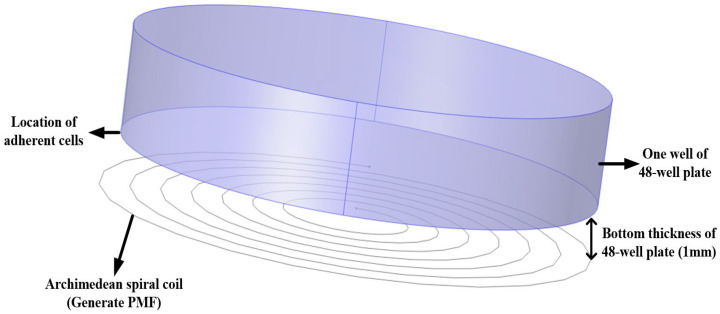
Schematic of simulation model.

Based on the above analysis and the actual size of the inductive coil, a model was established, as shown in Figure 9. The coil served as the source of the μs-PMF generation. Positioned 1 mm above the coil is a cylindrical model representing one well of the 48-well plate. The bottom of the cylinder is the location of adherent cells.

### 2.5. In Vitro Cell Experiment

The experiment encompassed three components: the toxicity experiment on IONPs, the experiment on the μs-PMF treatment, and the experiment on MMT.

#### 2.5.1. Toxicity Experiment

**Figure 10:**
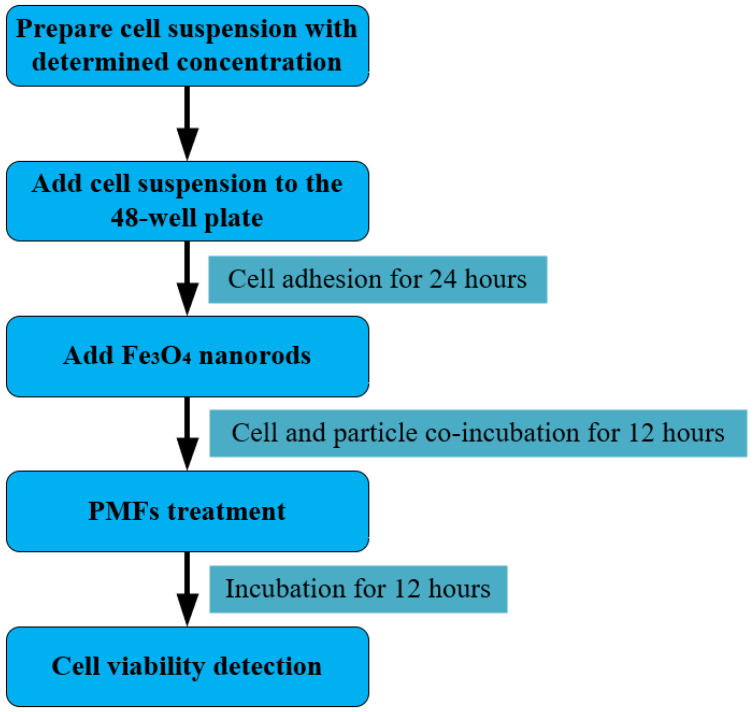
Schematic of MMT process.

The process of MMT is shown in the Figure 10. From the addition of MNPs to the detection of cell viability, cells and MNPs coexisted for 24 h. Therefore, this study explored the 24 h toxicity of IONPs.

As described in Section 2.3, in order to verify the theory between the μs-PMF and MNPs, four kinds of IONPs were selected for MMT, including nanospheres of three different sizes and nanorods of a single size. Among them, nanorods were determined under the guidance of the theory as the most suitable MNP for MMT with the μs-PMF. Consequently, the concentrations of the four types of IONPs were determined based on the toxicity detection results of the nanorods.

According to the relevant literature, the concentration of IONPs is generally set at 10 to 100 μg/mL [15,26,56,57,58,59,60,61]. Therefore, three concentration gradients were set for toxicity detection, namely 50 μg/mL, 100 μg/mL, and 150 μg/mL.

#### 2.5.2. μs-PMF Treatment Experiment

When A375 cancer cells filled 80% of the bottom of the culture bottle, they were digested and taken out. The cell count plate was used to determine cell concentration. Then, a certain amount of medium was added to the cell suspension, in order to dilute it to 5 × 10^4^ cells/mL. A pipette was used to blow the cell suspension evenly.

Prepared cell suspension of 300 μL was added to the 48-well plate. The plate was gently shaken in order to distribute the cells as evenly as possible. After being placed in the incubator for 24 h, the plate was taken out. Then, the old medium was sucked out. After the plate was cleaned twice with PBS, new medium of 300 μL was added. The plate was placed back into the incubator for 12 h again. Then, it was taken out and exposed to μs-PMF for 20 min. Finally, it was placed back in the incubator for 12 h before conducting viability detection.

#### 2.5.3. MMT Experiment

The process of the MMT experiment is shown in Figure 10, and is basically consistent with Section 2.5.2. The difference is that after the plate was placed in the incubator for 24 h, a new medium containing IONPs, with a concentration of 100 μg/mL, was used. Additionally, the cancer cells then coexisted with IONPs for 12 h before being exposed to the μs-PMF.

### 2.6. Cell Viability Detection

CCK-8 reagent was used to detect cell viability. After 12 h of exposure to μs-PMF, the old medium was sucked out. New medium of 220 μL containing CCK-8 reagent was added to the wells (the ratio of CCK-8 reagent to medium is 1:10). Then, the plate was placed back in the incubator for 2.5 h. Ultimately, its absorbance was detected by using an enzyme-linked immunosorbent assay (EPOCH2, Biotek, Burlington, VT, USA). In order to calculate the detected OD value as cell viability, two additional reference groups needed to be set up. One was the blank group with only the culture medium (containing CCK-8 reagent). The other was the control group with only cells (without IONPs and without μsPMF). The cell viability of the experimental group was calculated based on the OD values of the blank group, control group, and experimental group. The specific calculation method is shown in Equation (5).

(5) $$Cell\;viability=\frac{OD_{Experimental\;group}-OD_{Blank\;group}\;}{OD_{Control\;group}-OD_{Blank\;group}}\times 100\%$$

### 2.7. Data Processing and Statistical Analysis

All experiments were repeated at least five times independently, and the experimental data was statistically analyzed using Origin 8.0. The data was represented by mean ± standard deviation (mean ± SD), and a one-way Analysis of Variance (ANOVA) was used to evaluate the statistical differences between experimental data. *p* >0.05 represents no statistical differences between groups, * *p* < 0.05 represents statistical differences between groups, ** *p* < 0.01 represents significant statistical differences between groups, and *** *p* < 0.001 represents extremely significant statistical differences between groups.

## 3. Results

### 3.1. Magnetic Field Simulation

**Figure 11:**
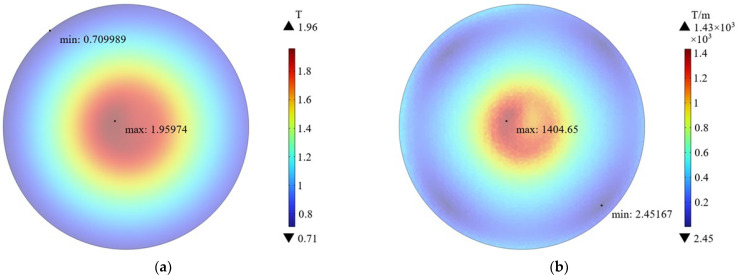
Schematic of magnetic field simulation results. (**a**) Magnetic flux density (T); (**b**) magnetic field gradient (T/m).

The magnetic field was simulated under the peak parameter of current (1640 A), and the results are shown in Figure 11a. The magnetic field shows a spatial distribution of weakening from the center to the edge, with the strongest magnetic flux density at the center of the coil reaching 1.96 T. The weakest point was at the edge of the coil, approximately 0.71 T. By calculation, the average was about 1.22 T.

Due to the uneven spatial distribution of the magnetic field, there exists a certain gradient. It was simulated and the results are shown in Figure 11b. The distribution of gradient was similar to the magnetic flux density, strong center, and weak edge. The gradient was 1404 T/m at the strongest position and 2.45 T/m at the weakest position. By calculation, the average was about 317 T/m.

### 3.2. MNPs Toxicity Detection

**Figure 12:**
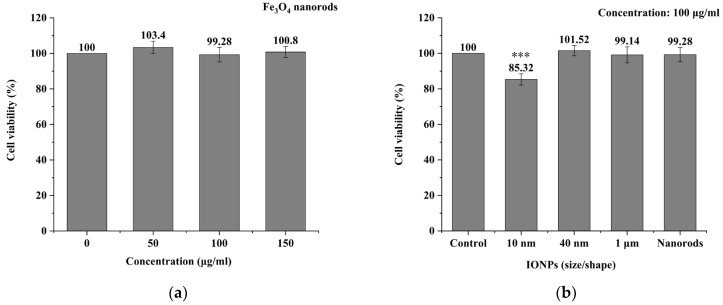
Toxicity detection results of IONPs. (**a**) Toxicity detection results of Fe_3_O_4_ nanorods at three concentrations (50 μg/mL, 100 μg/mL, and 150 μg/mL); (**b**) toxicity detection results of all four IONPs at a concentration of 100 μg/mL. *** *p* < 0.001.

Figure 12a shows the 24 h toxicity detection results of IONPs (nanorods) at three concentrations, 50 μg/mL, 100 μg/mL, and 150 μg/mL. The statistical analysis showed that there was no significant difference in cell viability between the experimental group and the control group (*p* > 0.05). This indicates that the nanorods have no significant toxicity to A375 cancer cells at these three concentrations. In some articles, when nanoparticles are used for in vitro cell experiments, their concentration generally does not exceed 100 μg/mL [15,26,56,57,58,59,60,61]. Therefore, the concentration of nanorods for the MMT experiment was preliminarily set to 100 μg/mL.

Subsequently, toxicity experiments of the other three IONPs (nanospheres) were carried out. Figure 12b shows the 24 h toxicity detection results of all four IONPs at a concentration of 100 μg/mL. Comparing the experimental groups with the control group, it was found that only the cell viability of the experimental group (10 nm) was statistically different from the control group (*** *p* < 0.001), while the other experimental groups (40 nm, 1 μm, and nanorods) were not statistically different from the control group (*p* > 0.05). This indicates that the toxicity of nanospheres (10 nm) reduce the viability of A375 cancer cells to about 85%, while the other three IONPs (40 nm, 1 um, nanorods) have no obvious toxic effect on A375 cancer cells. According to the literature, toxicity level is acceptable if it does not reduce cell viability below 80%.

In summary, the concentration of the four IONPs was set to 100 μg/mL.

### 3.3. μs-PMF Treatment and MMT

**Figure 13:**
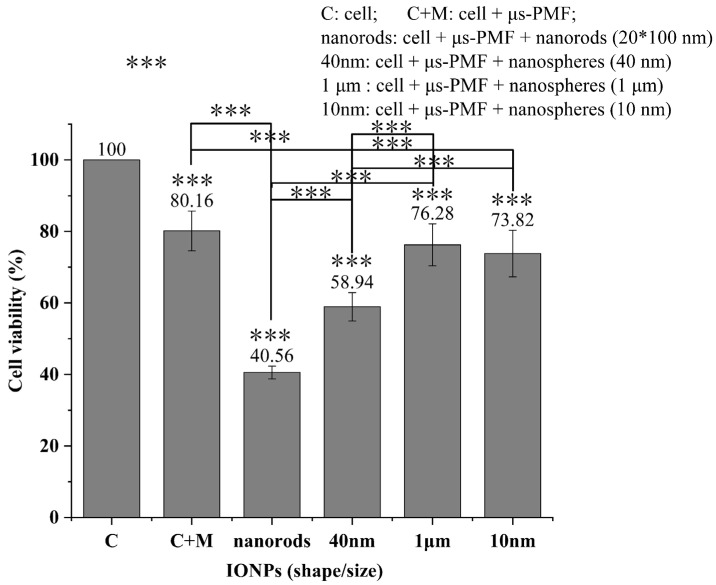
Results of μs-PMF treatment and MMT. *** *p* < 0.001.

Figure 13 shows the viability detection results of μs-PMF treatment alone and MMT. Specifically, Group C is the control group 1 where the cells were not treated; Group C+M is the control group 2 where the cells were treated by μs-PMF; Group Nanorods is the experimental group 1 where the cells were treated by μs-PMF and IONPs (nanorods); Group 40 nm is the experimental group 2 where the cells were treated by μs-PMF and IONPs (40 nm); Group 1 μm is the experimental group 3 where the cells were treated by μs-PMF and IONPs (1 μm); and Group 10 nm is the experimental group 4 where the cells were treated by μs-PMF and IONPs (10 nm).

According to Figure 13, the killing rate on cancer cells of Group C + M was 19.4%, Group Nanorods was 59.44%, Group 40 nm was 41.06%, Group 1 μm was 23.72%, and Group 10 nm was 26.18%; all with extremely significant statistical differences (*** *p* < 0.001).

Comparing Groups 10 nm, 40 nm, and 1 μm, it was found that the killing rates of the smallest IONPs (10 nm) and the largest (1 μm) were low, while the IONPs (40 nm) were the highest. Similar results were found in the literature [18]. We will explain this from the first and second parts of the matching theory, and the specific analysis was carried out in Section 4.2.

Comparing experimental groups (nanorods, 40 nm, 1 μm, and 10 nm) with Group C+M, it was found that Group Nanorods, which met the requirements of the theory, had the strongest killing effect, producing an additional 39.6% killing rate than the treatment of μs-PMF alone, and a killing rate at least 18.38% higher than that of the other three IONPs (10 nm, 40 nm, and 1 μm).

## 4. Discussion

### 4.1. μs-PMF Treatment

In Group C+M, the μs-PMF treatment alone had a 19.4% killing rate on cancer cells, possibly due to the high spatial gradient of the μs-PMF. Relevant articles have shown that a magnetic field with a parameter of 1300 T^2^/m can cause obvious morphological changes in osteoblasts [62], and with a parameter of 64.8 T^2^/m, can inhibit the proliferation of leukemia cells [63].

The parameters of the μs-PMF are shown in Figure 11. In the center, the gradient (∇B) is up to 1400 T/m and the magnetic flux density (B) is up to 1.96 T, so B∇B is 2744 T^2^/m, greater than the above. Zablotskii et al. calculated that a magnetic field of 10^3^ T^2^/m can produce stress of several Pa on cells, enough to affect cell function and morphology [64]. Therefore, we speculated that the decrease in cell viability in the Group C+M was due to the high spatial gradient of μs-PMF, which exerted magnetic gradient force on cells, affecting their function and morphology, and thus decreasing their viability.

### 4.2. Feature-Matching Theory of MMT

The MMT in this article was based on the μs-PMF and the proposed feature-matching theory. In order to further verify the guiding role of it in MMT, the results in Section 3.3 will be divided into three parts for further discussion, according to the three points of matching in the theory.

#### 4.2.1. MNP Volume and Brownian Relaxation Critical Volume

This part mainly discusses the differences between Group C+M, Group 40 nm, and Group 10 nm.

Comparing Group 40 nm with Group C+M, it was found that the cell viability of Group 40 nm was 21.22% lower than that of Group C+M, with an extremely significant statistical difference (*** *p* < 0.001). This indicates that the μs-PMF combined with IONPs (40 nm) produced an additional 21.22% killing rate than the treatment of μs-PMF alone. According to Section 2.3, the relaxation process of IONPs (40 nm) is dominated by Brownian relaxation, in which IONPs rotate and generate torque under the action of μs-PMF [35,36], and finally exert MMF on cancer cells. This force can be calculated by Equations (3) and (6), and is about hundreds of pN, which can cause damage to cells [42,65,66].

Comparing Group 10 nm with Group C+M, it was found that the cell viability of Group 10 nm was 6.34% lower than that of Group C+M, but without a significant statistical difference (*p* > 0.05). This indicates that the μs-PMF combined with IONPs (10 nm) did not have an additional killing effect compared to μs-PMF treatment alone; that is, IONPs (10 nm) hardly play a role in MMT paired with the μs-PMF. According to Section 2.3, the relaxation process of IONPs (10 nm) is dominated by Neel relaxation, so the mechanical rotation is very weak [35,36], resulting in almost no torque to exert force on the cancer cells. Although IONPs (10 nm) undergo oscillatory movement due to the high spatial gradient and temporal variation of the μs-PMF [11], the literature indicates that this oscillation is less effective than the torque in MMT [3]. In addition, some achievements have been made by using IONPs oscillations, but the frequency of their magnetic fields is generally above tens to hundreds of Hz [18,67], which is much higher than the 1 Hz of the μs-PMF. Therefore, it is difficult for IONPs (10 nm) to play a role with this μs-PMF.

To sum up, the μs-PMF (low frequency but high field intensity) developed in this study cannot fully utilize the oscillation of MNPs for MMT, and it is more suitable for combining with MNPs whose relaxation process is dominated by Brownian relaxation, so that the MNPs can rotate and exert MMF of hundreds of pN on the cells. This proves the correctness of the first part in the theory, “matching between MNP volume and the Brownian relaxation critical volume”.

#### 4.2.2. MNP Relaxation Time and the Pulse Width

This part mainly discusses the differences among Group C+M, Group 40 nm, and Group 1 μm. Group 40 nm is the same as in Section 4.2.1, so this section will not go into details.

Comparing Group 1 μm with Group C+M, it was found that the cell viability of Group 1 μm was 3.88% lower than that of Group C+M, without a significant statistical difference (*p* > 0.05). This indicates that the μs-PMF combined with IONPs (1 μm) did not have an additional killing effect compared to μs-PMF treatment alone; that is, IONPs (1 μm) hardly play a role in MMT paired with the μs-PMF.

Comparing Group 40 nm with Group 1 μm, it was found that the cell viability of Group 40 nm was 17.34% lower than that of Group 1 μm, with an extremely significant statistical difference (*** *p* < 0.001). This indicates that Group 40 nm had a better killing effect than Group 1 μm.

According to Section 2.3, the relaxation process of IONPs (40 nm and 1 μm) is dominated by Brownian relaxation, so that they can rotate and exert MMF on the cell under the action of magnetic field [35,36]. Moreover, according to Equations (3) and (6), the larger the size of the IONPs, the greater the force generated, and, theoretically, the stronger the killing effect. However, the results showed that the killing effect of the smaller IONPs (40 nm) was stronger than that of the larger (1 μm), and similar results were obtained in the literature [18,19].

These results can be explained by the mismatching between the relaxation time of the MNPs and the pulse width of the μs-PMF. According to Section 2.3.2, the relaxation time of IONPs (40 nm) is about tens of microseconds, which is less-than or close-to the pulse width of the μs-PMF. Therefore, without considering the influence of some other factors, in one cycle, IONPs (40 nm) can basically complete the rotational motion and fully exert MMF on cancer cells. Meanwhile, the relaxation time of IONPs (1 μm) is much larger than the pulse width of 28 μs, reaching hundreds of milliseconds. Therefore, under the action of the μs-PMF, there is not enough time for IONPs (1 μm) to complete Brownian relaxation, making their rotational motion very weak, and thus hardly exerting MMF on the cancer cells.

In summary, although the μs-PMF developed in this study has the advantages of high field intensity and high spatial gradient, it also has the disadvantages of narrow pulse width. Therefore, in order to enable the μs-PMF and the MNPs to play a full role in the MMT, the Brownian relaxation time of MNPs must in an order of or less than the pulse width. This proves the correctness of the second part in the theory, “matching between relaxation time of MNPs and the pulse width of the μs-PMF”.

In addition, the comparison between Group 10 nm and Group 1 μm showed little difference in cell viability, both of which were higher than that of Group 40 nm. According to Section 4.2.1 and Section 4.2.2, the rotational motion of both these two IONPs (10 nm and 1 μm) under the action of μs-PMF is too weak to exert MMF on cancer cells, which leads to their weak killing effect and similar cell viability.

#### 4.2.3. MNPs Shape and the Intermittence of PMF

This part mainly discusses the differences among Group C+M, Group 40 nm, and Group Nanorods. Group 40 nm is the same as in Section 4.2.1, Group Nanorods is the same as in Section 3.3, so this section will not go into details.

Comparing Group 40 nm with Group Nanorods, it was found that the cell viability of Group 40 nm was 18.38% higher than that of Group nanorods, with an extremely significant statistical difference (*** *p* < 0.001). This indicates that Group nanorods had a better killing effect than Group 40 nm.

According to Section 2.3.2, the volume of the two IONPs (nanospheres with a diameter of 40 nm, and nanorods with a length of 100 nm and a diameter of 20 nm) was basically the same, both were about 3 × 10^−23^ m^3^. Their relaxation processes were dominated by Brownian relaxation, and the relaxation time was also close to the pulse width. That is, both IONPs satisfy the first and second parts of the theory. Therefore, it is speculated that the difference in killing rates lies in their shape properties.

These two IONPs exert MMF on the cells mainly through the rotation caused by Brownian relaxation. Moreover, some articles have shown that the rotation of rod-shaped IONPs can have a stronger effect on cells than that of spherical IONPs, thus achieving a stronger killing effect [48,49,50]. This is consistent with the results in this study. Because of the periodicity and intermittency of the μs-PMF, the IONPs rotate repeatedly and are able to exert force on the cancer cells more times.

In summary, rod-shaped MNPs are more suitable for the μs-PMF, which also proves the correctness of the third part in the theory, “matching between MNP shape and the intermittence of PMF”.

### 4.3. Mechanism of MMT

Four kinds of IONPs were selected for MMT. Among them, IONPs (nanorods) are the most suitable IONP determined by the theory. Therefore, this section will conduct a specific analysis on the response of the nanorods to the μs-PMF, thus clarifying how the MMT has a killing effect on cancer cells. In addition, rod-shaped particles exhibit magnetic anisotropy due to their asymmetric shape, resulting in a direction with the lowest magnetization energy. The magnetic moment tends to be oriented in the direction with the lowest magnetization energy, which is called the direction of easy axis. The long axis direction of rod-shaped MNPs is the direction of their easy axis [41,51], so in the following analysis, the long axis direction of the nanorods was selected as the direction of their magnetic moment.

**Figure 14:**
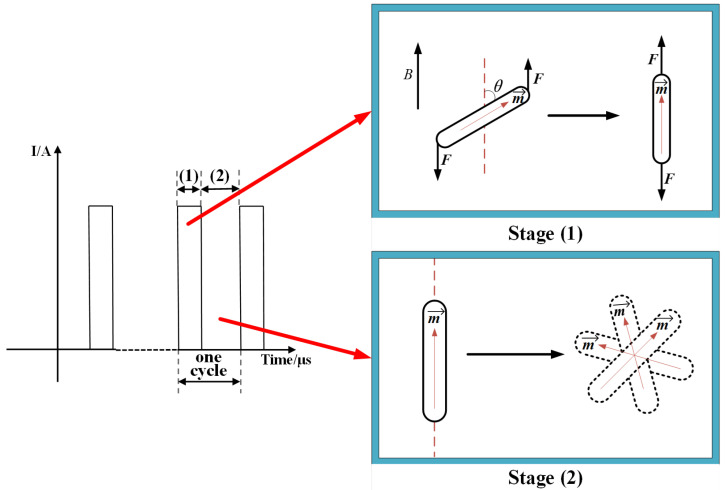
Schematic diagram of two stages of μs-PMF.

As shown in Figure 14, each cycle of μs-PMF can be divided into two stages, stage (1) with magnetic field, and stage (2) without magnetic field.

#### 4.3.1. Stage (1) with Magnetic Field

Before the μs-PMF took effect, IONPs (nanorods) were in a disordered state due to thermal motion, and their easy axes were arranged in any direction. When the magnetic field pulse was applied, as shown in the Figure 14, the nanorods had a deviation angle between their easy axis and the direction of the magnetic field, due to their previous disordered state. Then the nanorods underwent a process of Brownian relaxation. In this process, they were subjected to a torque of magnetic force, causing the easy axis to approach the direction of the external magnetic field, resulting in rotation until the direction of the easy axis was close to the direction of the magnetic field (due to the existence of thermal motion, the easy axis direction was not completely parallel to the direction of magnetic field). During this rotation process, as the particles had already targeted the cancer cells, the torque exerted on the nanorods also acted on the cells, causing damage. The formula for calculating the magnitude of this torque is shown in Equation (2), and the relationship between torque and force is shown in Equation (6). Through Equations (2) and (6), it was calculated that under this torque, the nanorods exert a force of about 200 pN on the cells. The relevant literature shows that a force of tens of pN is sufficient to cause damage to the cell membranes, organelles, and other structures [42,65,66], so this force is sufficient to cause injury or death to cancer cells.

(6) $$L=\frac{1}{2}Fl\sin\theta$$

here *F* is the magnetic force that generates torque; *l* is the length of the long axis of the nanorod (or the diameter of the nanosphere), approximately 100 nm; and *θ* is the angle between the long axis of the nanorod and the direction of the magnetic field.

#### 4.3.2. Stage (2) without Magnetic Field

In this stage, IONPs (nanorods) were no longer subjected to torque of magnetic force and no longer exerted force on the cancer cells. As shown in the Figure 14, the nanorods return to a disordered state of thermal motion, with their magnetic moments being arranged in any direction.

The whole process lasted for 20 min, during which the two stages alternated. This caused the nanorods to switch 1200 times between magnetization rotation and irregular thermal motion, thus exerting MMF (about 200 pN) on the cancer cells 1200 times. Finally, the cancer cells died by apoptosis or necrosis under this force.

### 4.4. Limitations of Feature-Matching Theory

In this paper, a feature-matching theory between PMFs and MNPs is proposed, which can guide the design of MNPs and the development of a PMF generator that can be used in MMT. In the process of proposing this theory, reference was made to the analysis ideas in the literature [37,38,68], so the influence of some factors was simplified. This study used strongly anisotropic magnetic particles in a viscous carrier liquid [69,70]. Therefore, only a preliminary consideration was given to the most important influencing factors in practical situations, such as the influence of MNP size on relaxation mechanisms and relaxation time [35,36,71,72].

But there is no doubt that the practical situation is always more complicated than the theoretical analysis. There are some other factors that were not been considered in this paper, such as the influence of environmental solution viscosity and the impact of interactions between MNPs on the relaxation mechanism. Although these factors may have a certain impact on the relaxation mechanism and relaxation time of MNPs, they will not cause fundamental differences in the matching theory; that is, MNP size still needs to be greater than the critical size in order for the Brownian relaxation mechanism to dominate [73,74,75]. The relaxation time of MNPs should be close to the pulse width of the PMF in order to enable MNPs to complete rotational motion.

To sum up, there are still some limitations in the application of this matching theory, which requires the use of strongly anisotropic magnetic particles in a viscous carrier liquid, and the weak interaction between MNPs. However, as more and more scholars pay attention to MMT, these factors will also be gradually taken into account, and the matching theory will become increasingly accurate.

## 5. Conclusions

In order to facilitate the application of a PMF in MMT, this article developed a μs-PMF generator. Based on its narrow pulse width and other properties, a matching theory between PMFs and MNPs is proposed for the first time, in order to guide the determination of their parameters in MMT. Four kinds of MNPs were selected for in vitro cell experiments, namely, Fe_3_O_4_ nanospheres (diameter of 10 nm, 40 nm, and 1 μm) and nanorods (diameter of 20 nm, length of 100 nm), in which nanorods fully meet the requirements of the theory. The results showed that the killing rate of μs-PMF with nanorods was 39.6% higher than that of μs-PMF alone, and at least 18.38% higher than that of μs-PMF with the other three IONPs, which did not meet the requirements of the theory. This proves the effectiveness of μs-PMF in MMT and the correctness of the theory.

In the future, the influence of other factors that were not considered in the matching theory should be further considered, including but not limited to the frequency of the magnetic field, dipolar interaction, and the elastic response of the cell membrane. This is of great significance for further promoting the clinical application of PMFs in MMT.

## Data Availability

The data presented in this study are available in this article.
